# Agenda-setting in policies related to high-risk sexual behaviours, stimulants, and alcohol abuse in Iranian adolescents

**DOI:** 10.1186/s12961-023-01050-1

**Published:** 2023-10-09

**Authors:** Saeid Mirzaei, Mohammad Hossein Mehrolhassani, Vahid Yazdi-Feyzabadi, AliAkbar Haghdoost, Nadia Oroomiei

**Affiliations:** 1 Non Communicable Diseases Research Center, Bam University of Medical Sciences, Bam, Iran; 2 Medical Informatics Research Center, Institute for Futures Studies in Health, Kerman University of Medical Sciences, Kerman, Iran; 3 Health Services Management Research Center, Institute for Futures Studies in Health, Kerman University of Medical Sciences, Kerman, Iran; 4 Health Modeling Research Center, Institute for Futures Studies in Health, Kerman University of Medical Sciences, Kerman, Iran

**Keywords:** Agenda-setting, High-risk behaviours, Stimulants abuse, Alcohol abuse, Adolescents, Health policy, Kingdon’s multiple streams framework, Iran

## Abstract

**Background:**

This research article retrospectively analyses the agenda-setting approach of policies concerning high-risk sexual behaviours, stimulant and alcohol abuse among Iranian adolescents.

**Methods:**

This qualitative case study policy analysis involved analysing 51 national documents and conducting interviews with 49 policy-makers and executives. Purposive sampling with a snowball strategy and semi-structured interviews were used. The data was analysed using the framework analysis method, with Kingdon’s multiple streams framework serving as the analytical framework.

**Results:**

The study has identified the confluence of several factors, including the problem stream, the policy stream and the political stream. Within the problem stream, several factors contributed, such as the prevalence of high-risk behaviours, strong scientific evidence on these behaviours, changes in human immunodeficiency virus (HIV) transmission patterns, increased statistics of poisoning and deaths related to alcohol abuse, and the visit of Iran’s supreme leader to the slums of Mashhad city. The policy stream has two periods of denial and acceptance. The denial period includes considering these high-risk behaviours to be the consequences of western culture, emphasis on the religious aspects and sinfulness of these behaviours, resisting the prevalence of anomalous behavioural patterns, abstinence and religious obligation of chastity, and avoiding ethical corruption. The acceptance period includes adolescents training, fear messages, promotional and cultural activities, parent training, school staff training, providing psychiatric services for withdrawal, counselling and reference to receive specialized services. The political stream involves global attention towards non-communicable diseases and high-risk behaviours, and the significant impact of preventing these behaviours during adolescence on the health status of society. Also, the supreme leader’s attention to social harms, and the establishment of the National Committee for Prevention and Control of Alcohol, have played significant roles.

**Conclusions:**

While the problem stream helped to highlight the problem and increase policy-makers’ attention, the politics stream played a significant role. Despite international evidence on the effectiveness of training in sexual issues in reducing high-risk behaviours, it did not succeed in being added to the agenda. The policy stream was heavily influenced by ideology and the political parties in power, affecting evidence-based policy-making. In countries with an ideological approach, the political stream plays a vital role in setting problems on the agenda.

## Background

Adolescence, a pivotal phase from childhood to adulthood, shapes lasting behaviour patterns influenced by factors such as hormonal shifts and societal pressures. Such changes can lay the groundwork for issues, with risky behaviour standing out as a common concern. This behaviour encompasses various actions that can detrimentally affect physical, psychological and social well-being in the short and long run [1–3].

The rise of high-risk behaviours poses a notable public health threat and has gained recognition as a paramount societal concern among health policy-makers due to swift social changes [4]. By 2030, tobacco use is projected to result in 10 million annual deaths and illnesses, illustrating a significant impact [5]. Considering the physical, psychological and social repercussions of additional high-risk behaviours such as substance abuse, violence and risky sexual behaviour, the resulting harm will be significantly multiplied [6]. Although risky behaviours’ detrimental impacts affect all segments of society, adolescents face a heightened vulnerability. Consequently, a substantial proportion of those facing the repercussions of such behaviours in the future will be the present-day adolescents [4].

Faced with challenges and crises during adolescence, numerous youths adopt behaviours jeopardizing both their immediate and long-term well-being. Such behaviours encompass substance abuse (especially stimulants and alcohol), violence and risky sexual conduct. Research has demonstrated links between high-risk sexual behaviours, alcohol consumption and substance abuse [7–10]. For instance, a study exploring substance use trends in Generation Z and its association with high-risk behaviours revealed that adolescent substance use is predominantly linked to stimulants, while a relationship exists between substance and alcohol use and high-risk sexual behaviours [11].

Therefore, due to the importance of the issue, different countries have adopted policies to combat these behaviours [12]. Additionally, several studies have analysed policy formulation and implementation in this field [13–15]. For instance, in Sub-Saharan Africa, the incorporation of research into policies concerning adolescents has played a role in elevating the visibility of adolescent-related concerns on the agenda [16].

Similar to numerous other nations, Iran has instituted policies to address these behaviours due to their significance. Being a developing country grounded in Islamic laws, Iran prohibits romantic involvement with the opposite gender outside of marriage, in accordance with Islamic regulations, and such actions are subject to penalties [17].

In accordance with Islamic laws and the Iranian criminal code, the consumption of alcoholic beverages, referred to as “shorb-e-khamr” in Islamic jurisprudence, is prohibited and carries penalties. Likewise, Iranian regulations prohibit the acquisition, trading and utilization of drugs and stimulants, with violations subject to punishment [18]. Even though sexual activity outside of marriage, alcohol consumption and drug abuse are categorized as offences in accordance with Iranian laws [19], studies highlight a growing prevalence of these behaviours within Iranian society, particularly among adolescents[20].

Agenda-setting is the process of elevating certain topics onto the policy agenda out of a multitude of possible issues that have garnered attention from policy-makers. Grasping this agenda-setting process holds significance in appreciating how various factors, groups and influences interplay [21]. Agenda-setting, akin to other phases of policy-making, is not an isolated process and is shaped by the subject matter, stakeholders, organizational communications and specific socio-political elements. Consequently, recognizing pivotal events and influential factors in introducing subjects to the policy agenda can enhance our comprehension of policy evolution and the contextual prerequisites for incorporating topics into the policy agenda [22].

Although several studies have examined the prevalence and determinants of high-risk sexual behaviours and associated behaviours such as stimulant and alcohol abuse among Iranian adolescents, to the best of our knowledge, no studies have employed an agenda-setting approach in this area. Identifying the agenda-setting approach could provide a valuable perspective for future policy-making aimed at controlling these behaviours in Iran and other countries facing similar circumstances. This research article retrospectively analyses the agenda-setting approach of policies concerning high-risk sexual behaviours and stimulant and alcohol abuse among Iranian adolescents.

## Methods

This qualitative case study policy analysis involved analysing documents and conducting interviews with policy-makers and executives. The documents analysed included all high-level and national publications related to adolescent health, high-risk sexual behaviours, and stimulant and alcohol abuse in adolescents that were written by public organizations and made available to the public between January 1979 and March 2022. The research team employed a purposive sampling method to identify key high-level documents related to health, adolescents, high-risk sexual behaviours, and stimulant and alcohol abuse in adolescents. The documents were obtained through library searches and by referring to the websites of various public organizations including the Ministry of Education, Ministry of Welfare and Social Affairs, Ministry of Health, Welfare Organization, Supreme Council for Cultural Revolution, Presidential Organization, Expediency Council, Ministry of Interior, Judiciary, Parliament, Anti-narcotics Headquarters, Police Force, Islamic-Iranian Model Progress Center, Education Organization, Justice Organization, General Department of Welfare of Kerman Province, Kerman University of Medical Sciences, Anti-narcotics Headquarters and Iran Statistics Organization. The documents were selected based on Jupp’s quadruple considerations, including authenticity (being original and genuine), credibility (accuracy), representativeness (being representative of the totality of the documents in their class) and meaning (what they say) [23]. In total, 51 documents were examined [24–74]. Each of these documents was studied separately and thoroughly by members of the research team. The relevant parts of the documents were selected, and the content of these parts was analysed. The results of the document analysis were then used to complement and verify the information gathered from the interviews.

Furthermore, it is important to highlight that the interdependence of our qualitative methods, involving both document analysis and interviews, presents a challenge in isolating codes and sub-codes by their individual sources. As our analysis process inherently integrates insights from these sources, attempting to segregate the codes and sub-codes according to their origins is not feasible without compromising the holistic representation of our findings.

Interviews were conducted with key informants who participated in the formulation, decision-making, policy development and implementation of policies related to these behaviours in adolescents to understand the events, streams and interactions that led to the inclusion of these issues on Iran’s policy agenda. The research team identified the initial interviewees by reviewing documents and consulting relevant organizations. Subsequently, each interviewee was requested to introduce key informants within this field.

The informants included all policy-makers and executives involved with these adolescent behaviours at national, provincial and local levels (local level, which includes Kerman city). Study participants included key policy-makers and executives, who were selected based on the inclusion criteria. Sampling was carried out using a purposive sampling technique with a snowball strategy. Semi-structured interviews were conducted, and data saturation was achieved after 49 interviews (Table 1).

**Table 1:** Interviewees

Level	Executive position	Number
National	Experts and officials of the Social Harms Office of the Ministry of Education	2
	Experts of the Family and School Population Health Office of the Ministry of Health and Medical Education	2
	Mental, Social and Addiction Health Office of the Ministry of Health and Medical Education	3
	Experts and officials of the Social Harms Prevention Office of the Welfare Organization	2
	Officials of the Cultural and Preventive Office of the Anti-narcotics Headquarters	1
	Member of the Independent Committee of Anti-narcotics of the Expediency Council	1
Provincial	Experts and officials of the Social Harms in General Kerman Provincial Department of Education	3
	Experts of the Family and School Population Health Office of the Kerman University of Medical Sciences	2
	Experts of the Mental, Social and Addiction Health Office of the Kerman University of Medical Sciences	2
	Experts and officials of the Social Harms Prevention Office of the Welfare Organization of Kerman Province	2
	Social Deputy of the Police Force of Kerman Province	2
	Experts of the Social and Cultural Office of the Provincial Government of Kerman	2
	Experts of the Social Deputy of Offence Prevention of Judiciary of Kerman Province	2
	Experts of Cultural and Preventive Department of Coordination Council of Anti-narcotics Headquarters of Kerman Province	1
	Chairman of the Coordination Council of the Anti-narcotics Headquarters of Kerman Province	1
Local	School consultants of Kerman city	12
	School principals of Kerman city	4
	School teachers of Kerman city	3
	Experts and officials of the Social Harms Office of Kerman city schools	2

The inclusion criteria of policy-makers and executives included being informed about the policies related to these behaviours in adolescents; being involved in agenda-setting, planning, writing and executing the policies; and having a tendency to participate in the research.

The recorded information from each interview was transcribed word for word onto paper by two researchers (NO and SM) immediately after listening to it several times. Note-taking was also conducted simultaneously with recording to document the data throughout the research process. The selected texts and conducted interviews were carefully studied and analysed by the two research team members (NO and SM) to obtain a comprehensive understanding of the content. Sub-codes were identified, repeatedly studied and examined, and then categorized under the main codes. Any disagreements in identifying the sub-codes or codes were discussed and resolved in a meeting with the research team members. In this study, theory played a role in data analysis and was influenced by data analysis [75]. The framework analysis and the Kingdon framework were used for data themes analysis [76, 77], and if new emerged, they were also mentioned.

## Accuracy and robustness of data

To ensure credibility, the participants expressed their opinions about the agreement of findings. Additionally, the research team engaged in participatory thinking about the revealed topics at different stages of the study. Data was collected through interviews and document analysis. Confirmability of the research was ensured by preserving the documents at all stages of the study.

Factors that ensured confirmability included the researchers’ interest in the phenomenon under study, their engagement with the data for a prolonged period and their effort to seek others’ opinions. Furthermore, since the study was conducted by a team under the supervision and guidance of experts, its dependability and confirmability were verified [78].

To ensure transferability of the implemented interviews and identified codes, they were validated by the interviewees. To ensure data reliability, two methods were used for data collection: interviews and document review.

## Results

### Agenda-setting

The present research results, which were mainly the results of interviewing the key people and analysing the documents, were presented based on the Kingdon model (Table 2).

**Table 2:** Kingdon’s agenda-setting streams

Agenda-setting	
Main Category	Sub-category
Problem stream	High prevalence of high-risk behaviours and non-communicable diseases in adolescents, young people and adults Conducting numerous strong scientific studies in high-risk behaviours and social harms. Change acquired immunodeficiency syndrome (AIDS) transmission pattern from injecting transmission to sexual one Reference to risky sexual behaviours of adolescents and young people as a vital factor in the sexual transmission of AIDS in the National Strategic Plan for AIDS Control in Iran Reducing the age of first drug abuse according to rapid situational analysis studies from 1994 to 2014 by the University of Welfare and Rehabilitation Sciences in collaboration with the Welfare Organization. Increased statistics of alcohol poisoning and deaths Increased statistics adolescents crimes (crime of Gandi Street and murder of Afghan female child after being raped by Iranian adolescent) Iran’s supreme leader’s visit to the slums of Mashhad Poisoning by alcohol abuse in Rafsanjan and Sirjan cities Broadcast of different programs, including documentaries on social harms Negative feedback about the performance of the Education Organization in different meetings Reduced feedback of organizations to upstream officials during the years and exacerbation of the problem
Policy stream	Denial period: Considering the high-risk behaviours such as drugs and alcohol abuse and sexual relationships outside the framework of marriage as the consequences of western culture Emphasis on the religious aspects and sinfulness of these behaviours. Resisting the prevalence of anomalous behavioural patterns Showing the negative effects of western vulgar non-ethical culture Abstinence and religious obligation of chastity and avoiding ethical corruption Acceptance period: Adolescents training Fear messages Promotional and cultural activities Parent training School staff training Providing withdrawal of psychiatry services Counselling and reference to receive specialized services
Politics stream	Global attention to non-communicable diseases and high-risk behaviours and the important role of prevention of these behaviours in the adolescent age group on community health, specific attention of UNICEF, WHO and UNAIDS to change AIDS transmission pattern in Iran The attention of the supreme leader of Iran to social harm and determining 5 priorities in the harm’s domain Establishment of the National Committee for Prevention and Control of Alcohol
Opportunity window	Exacerbation of the problem and increase of public attention to high-risk behaviours and social harms International streams, AIDS pattern change Writing the Document of Alcohol Abuse The emphasis of the supreme leader of Iran on the reduction of high-risk behaviours and social harms Adolescent group: One of the best target groups to do preventive interventions

The codes and sub-codes presented in this table represent the combined outcomes of our analysis, incorporating insights derived from both document review and interviews with policy-makers and implementers. AIDS, acquired immunodeficiency syndrome

### Problem stream

According to Kingdon, policy-makers are drawn to problems through three mechanisms: indicators, events and feedback. These factors capture the policy-makers’ attention and prompt them to take action. The following matters have been explained in this context.

### Indicators

One of the factors that contributed to the formation of a problem stream in this domain was the availability of various indicators and reports related to the prevalence of high-risk behaviours, social harms and deaths resulting from them in adolescents and adults. A significant portion of these incidents could have been prevented. As a result, high-level decision-making positions in Iran became sensitized and attentive to this issue. This led to the formation of supportive positions and interventions to control these issues throughout the country. The most important sources that made this problem stream significant among policy-makers were various studies, such as reports related to human immunodeficiency virus (HIV) from the Ministry of Health, forensic medicine reports, prevalence studies of drug and stimulant abuse, monitoring of the status of social harms ordered by Iran’s supreme leader and reports from the Expediency Council. An interviewee stated that *“a strong research team was responsible for monitoring the social harms ordered by the supreme leader, and their findings were presented in reports. The results of their research indicated that the conditions were very poor.”* (I23).

Another factor that contributed to the creation of the problem stream was the status of HIV and sexually transmitted diseases in Iran. Sexual transmission is one of the ways in which HIV can be transmitted, and the number of cases has been increasing in recent years. The Third National Strategic Plan of AIDS Control in Iran highlighted that the sexual behaviours of adolescents and young people could potentially lead to the sexual transmission of HIV in the population if no action is taken [79]. The population of Iran is predominantly young, and recent studies have shown a significant prevalence of high-risk sexual behaviours among youngsters. There has been an increase in the number of identified cases of sexual transmission, which has raised serious concerns about the start of the third epidemic wave, i.e., HIV sexual transmission. However, sexual behaviour outside the framework of marriage is considered unacceptable in Iran and is punishable by law, which means that these behaviours often occur secretly in society. One interviewee said:

“…*The situation is very serious, and it is believed that punishment alone will deter individuals from engaging in high-risk behaviours. However, this approach may not be effective, particularly among adolescents and young people of this generation*.” (I21).

Regarding drug abuse, studies conducted by the University of Welfare and Rehabilitation Sciences in 2004 and 2007 in the form of rapid situation assessments revealed that the age of addiction in Iran has decreased. Additionally, the consumption pattern of opioids, stimulants and psychedelics has also increased. [80, 81]. Furthermore, the University of Welfare and Rehabilitation Sciences conducted a study on the prevalence of high-risk behaviours among individuals aged 15–25 years. The study found that adolescents who consume drugs have a higher prevalence of engaging in other high-risk behaviours compared with their peers [82]. The results of this study led to an increased level of sensitivity within the Ministry of Health and Welfare Organization towards the issue of high-risk behaviours.

Due to the fact that alcohol abuse is considered taboo in Iran and is punishable by law, there are no authentic published statistics available on this issue. In some cases, officials in this field have announced paradoxical statistics. Despite the lack of accurate statistics on alcohol consumption, it has been noted that there has been an increase in its use in Iran, which is a cause for concern. One interviewee mentioned:

“*There are many problems like this where statistics are not available, and suddenly after several years, they are revealed as big uncontrollable issues. I have always maintained that the statistics we have or hear about are only the tip of the iceberg that is out of the water, and we only see the iceberg when we hit it like the Titanic.”* (I26).

### Focusing events

Several focusing events caused attention to be drawn towards high-risk behaviours and social harm in the general public, particularly among adolescents. These events included Iranian officials’ visits to the slums of Mashhad, instances of large-scale alcohol poisoning in the cities of Sirjan and Rafsanjan (located in Kerman Province), an increase in adolescent offences, such as the murder of an Afghan female child by an Iranian adolescent and the broadcasting of programs on social harms by the Islamic Republic of Iran Broadcasting.

One event that brought more attention to the issue of social harm was the visit of Iran’s supreme leader to the slums of Mashhad. During this visit, he demanded an improvement in the situation of marginalization and an examination of social harm. This event garnered the attention of policy-makers and brought the issue into focus, resulting in the promotion of the Social Council of the Country to the Social Affairs Organization. The Social Council of the Country was established in 2001 to develop policies related to the prediction, prevention and resistance against social problems and harms, as well as to coordinate with other organizations.

In 2016, the supreme leader delegated the presidency of the Social Council of the Country to the president, and the council was promoted to the Social Affairs Organization with the aim of preventing social harm. This organization then reached out to several entities, including the Welfare Organization, Ministry of Health, Anti-narcotics Headquarters, Education Organization and Judiciary, to collaborate on designing and implementing effective policies and programs to address high-risk behaviours and social harm among adolescents and young adults in Iran.

*“We cannot say that attention was not paid to social harm, but after the supreme leader’s visit, it became very important. He ordered to organize this situation and asked for reports; in this way, the harm were brought into focus. Many senior managers visited there, and the topic of harm prevention became hot.”* (I29).

*“The visit of the supreme leader brought attention to the issue, and as a result, the Social Council of the Country was promoted to the Social Affairs Organization, which took charge of coordinating and examining the matter. The organization encouraged all related organizations to propose programs and solutions to tackle the problem, and subsequently, various plans were suggested by the Ministry of Health, Education Organization, and other entities….”* (I27).

The large-scale alcohol poisoning incidents in Rafsanjan and Sirjan cities in Kerman Province were the focusing events that brought attention to alcohol abuse in Iran. In 2013, 298 people were poisoned with alcohol in Rafsanjan, resulting in the death of 4 people. In 2017, a similar incident occurred in Sirjan, which led to the poisoning of 114 people and the death of 5 people. These incidents received widespread media coverage and raised awareness about the issue of alcohol abuse. Policy-makers began to pay more attention to this issue, which had previously been overlooked.

*"It made the policy-makers realize that the alcohol problem cannot be solved by whipping and fines alone, and that the number of cases is not limited. Therefore, they needed to take serious measures. For instance, how many people are living in Rafsanjan and Sirjan, and how many of them consume alcohol? Now, imagine a city like Tehran. This incident compelled policy-makers to think seriously about the problem."* (I5).

According to statistics provided by the Judiciary, offences committed by adolescents have increased significantly, and this has been occasionally mentioned in the media. However, two high-profile crimes on Gandi Street – the murder committed by two male and female adolescents in a love story – and the murder of an Afghan female child by an Iranian male adolescent after raping her, brought the issue of adolescents to the attention of policy-makers more than ever before.

*“The story of Gandi Street, which occurred in 1996, was a wake-up call that our adolescents need help, despite some who still deny it. At that time, some blamed the influence of cultural invasion from satellite television in their homes. Then, in 2016, the tragic story of the Afghan child who was raped and murdered by an Iranian male adolescent highlighted the fact that we have not adequately trained our adolescents to manage their instincts. We also perpetuate taboos by telling them to keep silent about sensitive issues….”* (I6).

Another event that brought attention to social harm was the broadcasting of programs and documentaries on the subject. After years of neglect, television networks began to focus on social harm, and their coverage attracted the attention of the public and policy-makers alike.

"In the 1990s, we had very good documentaries on social harm, such as *Hell but Cold* and *Days without Calendars*. These programs won prizes, but they were never broadcast on TV. They addressed issues such as adolescents, child labor, divorce, addiction, etc.; however, they were not aired on TV. In 2000, the media atmosphere improved, and as social problems continued to increase, a documentary called *Shock* was broadcast on TV. It was then understood that people must be made aware of these issues, demand change, and even help policymakers." (I9).

### Feedback

Regarding feedback, we can mention two points. The first one is that the feedback proposed about the Education Organization in different meetings, including the shared meetings with the Ministry of Health and the Social Council of the Country, showed a lack of satisfaction with the performance of the Education Organization in preventing the harm. An interviewee stated:

“The representative of Judiciary told him (the Education Organization) that their performance is satisfactory, but he did not know why their Correctional centre^1^ and prisons are exploding!” (I11).

The second issue in this regard is the feedback provided by organizations to higher officials. Most of the interviewees believed that one of the reasons for the worsening problems in adolescents is the secrecy and censorship of events by organizations, as they are concerned about being accused of negligence. *“one of the reasons for worsening problems in adolescents is the secrecy and censorship of events by organizations. These organizations are often concerned about being accused of negligence, so they only report their successes to upstream officials and withhold information about problems. As a result, problems continue to grow secretly and unchecked, until they become too big to ignore."* (I19).

### Policy stream

The policy stream refers to policy solutions and alternatives for solving significant problems. Generally, the policy stream about these behaviours and other social harms, from the beginning of the Iranian revolution until now, included two approaches: denial and acceptance.

During the denial period, policy-makers denied the existence of the problem and attributed all social harms and events to cultural invasion. Therefore, policy-makers considered the occurrence of these behaviours to be the consequence of western culture. The policies during this period were based on emphasizing the religious aspects of behaviours, resisting the prevalence of anomalous behavioural patterns, showing the negative effects of western vulgar non-ethical culture, promoting abstinence and emphasizing the legal and religious obligation to consider chastity and avoid ethical corruption.

During the acceptance period, different programs and solutions were proposed by various organizations to address the issue of social harms. Despite the range of programs proposed, all focused on life skills, social empowerment, parenting styles and increasing awareness among parents and education staff regarding high-risk behaviours. Fear messages targeting those over 15 years of age were also employed to prevent high-risk behaviours such as drug and alcohol abuse, and in cases where adolescents were struggling with substance abuse, they would be referred to a psychiatry centre for withdrawal. In the case of high-risk sexual behaviours, the abstinence approach was the dominant strategy adopted. Although the Ministry of Health proposed policy to provide training about sexual issues and pregnancy health in schools, it was not well received. *“We deny the existence of sexual topics greatly. The truth is that there are many instances of sexual relationships before marriage. In a study that has not yet been published, the numbers regarding sexual behaviours before marriage were quite high; however, we believe that the numbers are even higher in reality because it was a self-declaration study. In the general public, we think that the statistics are even higher, but we do not talk about it, and we haven’t talked about it for years. For instance, we have observed a changing pattern of HIV transmission from drug injection to sexual transmission, while some days ago, I heard on TV that HIV transmission in Iran only happens through drug injection. Denying the existence of this issue is a big mistake….”* (I36).

The interviewees suggested that there is no distinct boundary between denial and acceptance in Iran’s approach towards high-risk behaviours. They emphasized that this issue is significantly impacted by the political party in power during each period. Some parties acknowledge the problem and implement policies based on acceptance and problem-solving, while others choose to deny the issue and implement policies based on denial. "*We cannot definitively say that we have moved past the denial period. While the situation has improved compared to the past, it still heavily depends on the political party in power in Iran. It’s like a cycle; sometimes we deny and keep the problems hidden, and then the issues are brought to the forefront by a political shift, and we enter an acceptance period*." (I42).

### Politics stream

The global attention towards non-communicable diseases and high-risk behaviours, along with their significant impact on community health, has led to officials of the Ministry of Health seeking solutions to address these issues. Furthermore, the special attention given by organizations such as the United Nations Children’s Emergency Fund (UNICEF), WHO, and the United Nations Program on HIV/AIDS (UNAIDS) to the changing pattern of AIDS transmission in Iran, with signs of an increase in HIV sexual transmission, has caused serious concerns for the Ministry of Health officials. This has led to fears of the beginning of the third epidemic wave, i.e., the HIV sexual transmission epidemic in Iran. On the other hand, Iran’s supreme leader proposed a national division plan to the Ministry of Interior in the domain of social harm management. He determined five priorities to solve the problems, among which addiction, drug abuse and ethical corruption were mentioned. He demanded all organizations try an organized, scientific and integrated way to prevent social harm. The order of the supreme leader, as the highest political position in Iran, caused an appropriate opportunity for all relevant organizations to be more involved in reducing social harm. Some involved organizations in this regard included the Education Organization, Welfare Organization, Judiciary, Anti-Narcotics Headquarters, police force and Ministry of Health.

In 2013, the Ministry of Interior, in collaboration with the Ministry of Health and other organizations involved in the prevention, therapy and reduction of alcohol harm, established the National Committee for Prevention and Control of Alcohol. This committee played a vital role in creating the National Document of Prevention, Therapy, Reduction and Rehabilitation of Alcohol Harms, which provided a framework for actions against alcohol abuse and defined the roles and responsibilities of various organizations involved in addressing this issue. The Ministry of Health, Ministry of Education, Anti-narcotics Headquarters and Judiciary were the main policy entrepreneurs that benefited from this framework and set policies primarily focused on prevention.

## Discussion

### Policy-making window

The process of formulating policies related to high-risk sexual behaviours and stimulant and alcohol abuse in adolescents is influenced by various factors within the policy-making domain. In Iran, various indicators and reports have highlighted the prevalence of these behaviours among adolescents. This finding is consistent with the observation of Behzadifar et al., who noted that the presence of evidence and statistics showcasing the worsening of the current situation serves to capture the attention of policy-makers and leads to the inclusion of the policy on the agenda [83].

Additionally, negative feedback about the performance of education organizations and censorship of events by organizations have exacerbated the issue, contributing to the creation of a problem stream. Yanovitzky et al. also mentioned that indicators, feedback and focusing events played a vital role in bringing youth alcohol consumption to the agenda setting process[84]. Similarly, the findings of Cohn et al. revealed that media coverage of methamphetamine use and its consequences as a crisis over time played a significant role in bringing the issue to the agenda setting [85]. According to Queirolo et al., public opinion data reflecting concerns about public safety had an impact on the legislation regarding marijuana in Uruguay [86].

On the other hand, the international attention to high-risk behaviours, writing the Document of Prevention and Alcohol Abuse Therapy, concern about HIV sexual transmission and, the most important one, the special attention of Iran’s supreme leader to social harms domain provided the political stream so that the opportunity to accept the problem and presence of related policies about prevention and therapy of these behaviours in adolescents could be created. On the contrary, if the political stream does not align with the problem and policy streams, gaining entrance to the agenda-setting process could prove to be a challenging and perhaps impossible task. This was demonstrated in the case of the alcohol pricing policy in England. Hawkins and McCambridge’s findings showed that the alcohol pricing policy in England was influenced by public opinion. Although influential policy actors throughout the Scottish government supported minimum unit pricing, this policy did not receive comparable political endorsement in England [87].

Separate streams come together at a special time to identify a problem, provide a solution and create a political change that leads to an appropriate time to change policies. However, there are potential limitations, and the opportunity window to act is often short-lived [88]. In fact, these streams converge at a particular time when a policy-making window opens. This window is created when attention is drawn to problems or through the emergence of a political opportunity. In this scenario, a specific issue has the opportunity to transform into an alternative solution [89].

Social harm and high-risk behaviours have been ongoing problems in public, especially among adolescents. However, policy-makers have not implemented specific policies to address these issues, which indicates a lack of attention given to these problems on the policy-makers’ agenda. Without a solution to a problem, policy-making in the form of policies cannot be enacted [90]. As time passed, the problem of social harm and high-risk behaviours intensified, and with the increasing attention to this issue by the public and political opportunities such as the changing pattern of AIDS, the creation of the Alcohol Abuse Document and the emphasis of Iran’s supreme leader on the harms of these behaviours, the issue was finally set on the policy-makers’ agenda. The primary solution to this problem is prevention, which is one of the best ways to reduce the volume of harm. Therefore, prevention should be the main focus when addressing this problem, particularly in schools and for the target group of children and adolescents, to reduce the number of harmful incidents in the future. With this approach, the problem of high-risk behaviours among adolescents can be effectively addressed through prevention and therapy measures. The issue of social harm caused by these behaviours is considered a momentum phenomenon. It is important to note that, sometimes, the intersection of these streams can provide an opportunity for policy-makers to address the issue and take appropriate action [91]. The Ministry of Health, Ministry of Education, Anti-narcotics Headquarters and Judiciary acted as policy entrepreneurs by coordinating the different streams and bringing attention to the problem of high-risk behaviours in adolescents. They requested the Social Affairs Organization to address the issue, ultimately leading to the problem being set on the agenda.

A policy-making window can open through either attracting attention to a problem or through a political opportunity [92]. This creates an opportunity for a unique topic to be considered and potentially lead to policy changes [77]. Due to the increase in social harm, attention was drawn to the issue, and Iran’s supreme leader emphasized the prevention and reduction of social harm, with a special focus on addiction and social corruption. This led to the topic of high-risk sexual behaviours, stimulants and alcohol abuse in adolescents being put on the agenda for prevention and timely therapy of the harms. The strong political stream in this domain ensured that the topic remains on the agenda. Mirzaei et al. also highlighted the significant role of the political stream in setting the policy agenda for the treatment of substance use disorders in Iran and creating an opportunity window for policy change [93].

According to Kingdon’s multiple streams framework, even if a problem gains attention and a political opportunity arises, not all proposed policies may be implemented. In the case of the Ministry of Health’s proposal to implement sexual and pregnancy health training in schools, although the problem of high-risk sexual behaviours in adolescents was on the agenda, this specific policy proposal was not added to the agenda. This may be due to various factors, such as resource constraints or competing policy proposals [94]. In this regard, findings of Thow et al. showed that the acceptability of policy solutions plays a critical role in policy-making [95].

Although training in sexual issues was proposed as a solution based on evidence and experiences from other countries, it could not attract the value acceptability of policy-makers and the public.

This finding contradicts the results of Behzadifar et al., who noted that the presence of international evidence related to hepatitis C contributed to the inclusion of this policy on the agenda [96]. Liu et al. also mentioned that policy options that adhere to current policies and regulations are more likely to be selected over ones that rely on other criteria such as technical feasibility, and future constraints [97].

Kingdon emphasized the importance of values and ideological factors in defining problems and proposing solutions [76], a point that Kadt also made in his analysis of problem-solving frameworks [98]. Despite policies entering the acceptance period, a denial approach still exists in policy-making regarding high-risk sexual behaviour, with abstinence policy remaining the main policy in this field. Evidence suggests that there are three strategies for coping with high-risk sexual behaviours: abstinence, having not more than one sexual partner and using a condom [99]. However, policy-makers do not give attention to those who do not accept the abstinence policy and do not provide comprehensive and expanded training for those who follow the other two strategies. This could have a significant impact on the spread of the virus in the coming years, given the changing pattern of AIDS transmission towards sexual transmission in Iran [100]. Considering that Iran is a country governed by Islamic laws, where matters related to marriage and procreation are vital and emphasized aspects of the Islamic faith, providing sexual education starting from adolescence within the context of preconception and fertility health concepts could potentially pave the way for incorporating these policies into the agenda.

## Conclusions

The multiple streams approach takes into account different dynamics in problem statement, solution and political processes, which converge in an appropriate opportunity. In this study, each indicator item, feedback and focusing event was found to be influential in raising the problem. While the problem stream helped to highlight the problem and increase policy-makers’ attention, the politics stream played a significant role. The supreme leader’s involvement in establishing Social Affairs Organization and announcing priorities in the field of social harm helped to put this problem on the agenda.

The policy stream showed two periods of denial and acceptance, with policies mainly focused on prevention, abstinence and resisting drugs and alcohol, but not harm reduction, particularly in the domain of high-risk sexual behaviours. Despite international evidence on the effectiveness of training in sexual issues in reducing high-risk behaviours, it did not succeed in being added to the agenda. The policy stream was heavily influenced by ideology and the political parties in power, affecting evidence-based policy-making.

Overall, it appears that, in countries with an ideological approach, the political stream plays a vital role in setting problems on the agenda. Policy entrepreneurs can use various means, such as mass media, scientific studies and statistics, to draw attention to the issue and provide the possibility of setting the problem on the agenda.

## Data Availability

The datasets used/analysed during the current study are available from the corresponding author on reasonable request.

## Footnotes

1 Correctional centre is a place to keep the offenders under the age of 18 years.
